# Thromboelastogram and coagulation function index: relevance for female breast cancer

**DOI:** 10.3389/fonc.2024.1342439

**Published:** 2024-07-17

**Authors:** Qiongle Peng, Jinmei Zhu, Xiaoling Ren

**Affiliations:** ^1^ Department of Blood Transfusion, Affiliated Hospital of Jiangsu University, Zhenjiang, China; ^2^ School of Medicine, Jiangsu University, Zhenjiang, China; ^3^ Department of Medical Laboratory, Wuxi Traditional Chinese Medicine Hospital, Wuxi, China

**Keywords:** thromboelastogram, coagulation function, breast cancer, hemorrhagic diseases, hypercoagulability

## Abstract

**Introduction:**

Screening and postoperative intervention of breast tumors are critical for the effective diagnosis and treatment of disease development, and reliable diagnostic/screening methods become a key link.

**Objective:**

Thromboelastogram (TEG), routine platelet (PLT) count, and the coagulation function indicators in patients with different breast diseases were determined and analyzed to explore their predictive value in secondary bleeding disorders.

**Methods:**

A total of 131 patients with breast diseases, admitted to Jiangsu University Affiliated Hospital from January 2019 to December 2022, were selected as the research subjects. The detection items were analyzed using the receiver operating curve (ROC) after grouping for secondary bleeding disorders of patients with breast cancer.

**Results:**

The reaction (*R*) and the coagulation (*K*) times were lower in the malignant breast disease group, while the coagulation angle (*α*), maximum amplitude (MA), coagulation index (CI), fibrinogen (FIB), and D-dimer (D-D) were higher than those in the benign breast disease group. The *t*-tests proved that the MA and FIB values were statistically significant (*p* < 0.05) in the benign and malignant breast disease groups. The *R* and *K* in patients with breast diseases were positively correlated with the activated partial thromboplastin time (aPTT) and D-D, but were negatively correlated with PLT. The *α* angle was negatively correlated with aPTT and D-D, but was positively correlated with PLT. The MA for PLT function was positively correlated with FIB and PLT. CI was negatively correlated with aPTT, thrombin time (TT), and D-D, but was positively correlated with PLT. ROC curve analysis showed that the CI and *α* angle had a significant predictive value, whereas the correlation of the other indicators was relatively low.

**Conclusion:**

Coagulation tests showed significant differences in patients with breast cancer, differing from those with benign breast diseases. TEG combined with conventional coagulation indicators is potentially valuable for the prediction of secondary bleeding disorders in patients with breast cancer.

## 1 Introduction

Thromboembolic and hemorrhagic diseases caused by coagulation are some of the important causes of death in patients with malignant tumors. Armand Trousseau first described the connection between blood clots and cancer in 1865 (1). Since then, more research has discovered that patients with tumors are prone to developing hypercoagulable blood, and multiple studies support the interaction between tumor cells and the coagulation system in various ways (2). Cancer cells in the blood of patients with malignant tumors coagulate with other cells or with cancer cells themselves to form a thrombus, called cancer-related thrombus (cancer-associated thrombosis, CAT), which is one of the serious complications in patients with malignant tumors, and the fatality rate is high (3). Patients with malignant tumors are prone to venous thromboembolism (VTE) under a hypercoagulable state (4). A study on patients with colorectal cancer showed that the incidence of postoperative VTE in patients with malignant tumors is higher and lasts for more than 1 month, which is related to their hypercoagulable state (5). The incidence of cerebral venous thrombosis and visceral venous thrombosis (splanchnic vein thrombosis, SVT) is particularly high in patients with myeloproliferative tumors (myeloproliferative neoplasm, MPN) (6), despite the mechanisms of hematological malignancy differing from those of breast cancer (7). SVT includes Budd–Chiari syndrome and portal vein thrombosis (8). In addition, transitional thrombophlebitis, arterial thrombosis, disseminated intravascular coagulation (DIC), and non-bacterial thrombotic endocarditis (NBTE) have all been classified as complications of a hypercoagulable state in patients with malignant tumors (9). These studies provided proof of the complex relationship between blood hypercoagulability and various tumor diseases, with heavy family burden and economic challenges.

At present, research on the potential mechanisms of blood hypercoagulability in patients with malignant tumors mainly reflects the following aspects: a) tumor cells release procoagulant substances (10); b) the interaction between tumor cells and the fibrinolytic system (11, 12); c) tumor cell-mediated platelet (PLT) activation (2, 13); d) tumor-related complement activation (14, 15); e) genetic factors (16, 17); and f) clinical factors (18, 19). Increasing research on epidemiological and biological issues has presented the correlation between blood hypercoagulability and thrombosis, although the exploration of its mechanism is still on the way (20–22). For instance, circulating tumor cells are associated with an increased risk of VTE in patients with metastatic breast cancer (23). The dissemination of tumor-derived microvesicles promotes hypercoagulability and increased PLT activation (24). Intrinsic heparin plays an important role in balancing the blood flow patency and thrombosis (25). In cancer therapy, preoperative irradiation produces lesions triggering both bleeding and thrombosis, and most chemotherapeutic protocols affect PLT synthesis (20).

More studies have shown that the blood of patients with malignant tumors is in a state of hypercoagulability (26). The monitoring of the blood hypercoagulable state coupled with disease diagnosis generally involves clinical and laboratory examinations. In addition, the expert experience system based on histological examination and pathological analysis provides evidence for the preliminary diagnosis and further assessment of a disease (27). Laboratory thromboelastogram (TEG) (28, 29) and the blood coagulation function indices (20) indicate that patients with malignant tumors go through a state of blood hypercoagulability, which is significant for nonsurgical cancer therapy and preoperative irradiation. The TEG parameters of patients with tumors deviate from the normal value, and the blood is in a state of hypercoagulability (26). In the histological, anatomical observation of patients with malignant tumors, the thrombus of PLTs and the fibrin in blood vessels increased abnormally, and the immunohistochemistry of fibrin around tumor cells showed an increase of coagulation products, namely, PLT clot and fibrin (29). Immunohistochemical analysis is of value in the auxiliary diagnosis of malignant tumors, showing that histological examination provides evidence of a blood hypercoagulable state in clinical diagnosis (30). In terms of pharmacology, anticoagulants or anti-PLT drugs for patients with malignant tumors are useful for anticoagulation factors (31), indicating the blood hypercoagulable state in these patients, and effective intervention can assist the treatment. Clinical studies of several patients with MPN have shown that oral anticoagulants could reduce tumor metastasis and improve prognosis. The recurrence rate of VTE is fairly low, and there is no complication of massive hemorrhage (32).

**Figure 1:**
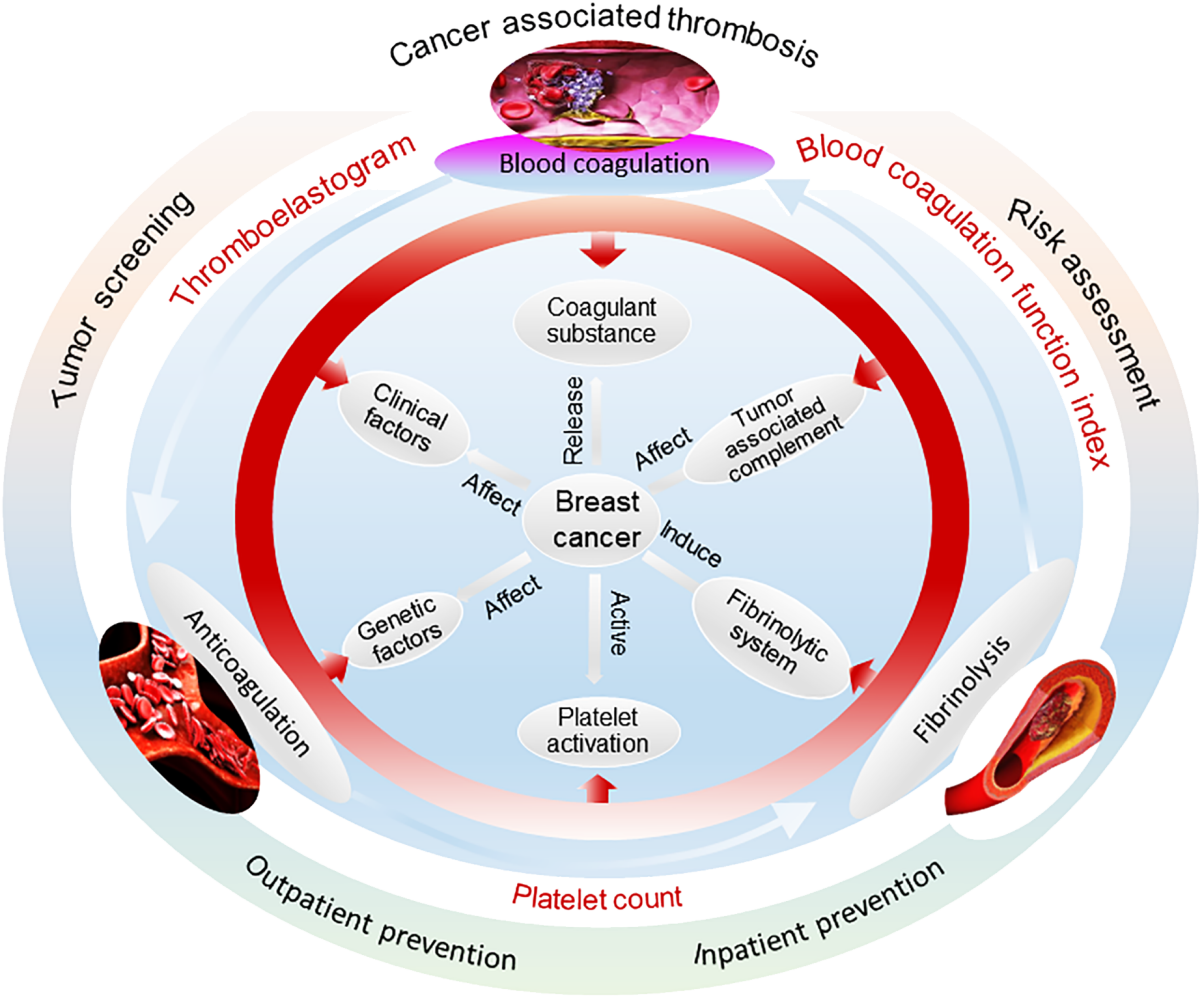
Mechanisms of the occurrence and development of breast cancer that relate to many factors from various aspects. Cancer-related thrombosis has become an important basis for breast cancer prediction and clinical analysis. Blood parameters of the thromboelastogram and routine blood and platelet count are important in providing effective information for medical interventions such as tumor screening, risk assessment, and inpatient and outpatient prevention.

Breast cancer is the most common malignant tumor endangering women’s health (33), and it is of great significance to explore the changes in coagulation function in patients with breast cancer. The hypercoagulable state of blood is closely associated with breast cancer-related thrombus and directly affects the results of breast tumor screening, risk assessment, hospitalization and hospitalization prevention, and other medical interventions. In biomedicine, breast cancer relates to complex systemic factors in many aspects, including clinical factors, genetic inheritance, coagulant substance, PLT activation, and tumor-associated complement (**Figure 1**). In this sense, cancer-related thrombosis has become an important basis for the prediction and clinical analysis of breast cancer (3). A number of blood parameters related to transfusion technology [e.g., TEG (34), routine blood test (35), and PLT count (36)] are important in providing effective information during the whole procedure of medical interventions, including tumor screening (37), risk assessment (38), and inpatient (39) and outpatient prevention (40). Research shows that venous thrombosis is one of the common causes of death in patients with breast cancer (41). TEG, as a laboratory indicator reflecting the dynamic changes of blood coagulation, presents an important guiding value for comprehensive analysis in combination with conventional indicators of coagulation function. The positive and negative predictive values can be determined using TEG for bleeding and thrombosis (42). Therefore, in this study, we examined and analyzed the changes in TEG, PLT count, and coagulation function in patients with breast cancer and in those with benign breast diseases. We assessed the risk of patients with breast cancer suffering from blood disorders by conducting a correlation analysis. It is hoped that the results of this study guide the prevention of such complications for the preliminary detection of breast cancer in clinical procedures.

## 2 Materials and methods

### 2.1 Samples

A total of 131 patients with breast diseases were gathered remotely from clinical facilities admitted to the Affiliated Hospital of Jiangsu University from January 2019 to December 2022 as the research subjects. These patients were aged 25–88 years and with complete case data. The exclusion criteria were serious heart failure, liver and kidney diseases, and blood system diseases, as well as the use of medication within a week that affects the coagulation function, such as anticoagulants and procoagulants (**Table 1**). According to the pathological diagnosis, patients with breast tumors were divided into two groups, including 53 cases of malignant breast cancer and 67 cases of benign breast tumor. After determining the TEG parameters, PLT count, and the coagulation function indicators, the two groups were compared for differences in each indicator. Furthermore, patients with breast cancer were divided into the secondary bleeding disorder group (nine cases) and the non-secondary bleeding disorder group (44 cases) according to their clinical manifestations. The receiver operating characteristic (ROC) curve was used to analyze the diagnostic value of the above detection items for the secondary bleeding disorder of patients with breast cancer.

**Table 1:** Inclusion and exclusion criteria during sample collection for this research.

Item	Discrimination	Inclusion	Exclusion
Breast disease	Pathological diagnosis	Yes	No
Age (years)	25–88	Yes	No
Gender	Women	Yes	No
Case data	Complete	Yes	No
Intravenous catheterization	Within a week	No	Yes
Anticoagulants	Within a week	No	Yes
Coagulants	Within a week	No	Yes
Cardiac insufficiency	With	No	Yes
Liver and kidney diseases	With	No	Yes
Hematological diseases	With	No	Yes

### 2.2 Examination methods

#### 2.2.1 Routine coagulation determination

The fasting venous blood of patients was collected in the morning. These samples were placed into a sodium citrate anticoagulant tube, fully mixed, centrifuged at 3,000 rpm for 15 min to separate plasma, and assessed within 2 h. The instrument used was a Sysmex CS-5100 fully automatic blood coagulation analyzer, which utilizes coagulation tests to determine the prothrombin time (PT), activated partial thromboplastin time (aPTT), thrombin time (TT), and fibrinogen (FIB). Immunoturbidimetry was used to determine the D-dimer (D-D). Routine coagulation function indices such as the PT and aPTT were also measured based on plasma (43).

#### 2.2.2 Platelet count determination

The fasting venous blood of patients was also collected in the morning and placed into an EDTA anticoagulant tube, fully mixed, and examined within 2 h. The instrument used was a Sysmex XE-2100 fully automatic blood cell analyzer.

#### 2.2.3 Thromboelastography determination

The fasting venous blood of patients was further collected in the morning and placed into a sodium citrate anticoagulant tube, fully mixed, and examined within 2 h. The instrument used was Haemonetics TEG 5000 (Haemoscope Corporation^®^, Niles, IL, USA), while the supporting kit was the activation coagulation detection kit (coagulation method) (TEG Hemostasis System Kaolin, Haemonetics Corp., Braintree, MA, USA). An activated coagulation detection reagent is a standardized reagent that includes kaolin, mixed phospholipids, and buffer stabilizers, which can detect coagulation disorders related to the intrinsic and extrinsic coagulation pathways. Kaolin activators are similar to diatomaceous earth, but are not easily affected by aprotinin. In this study, the kaolin reagent was firstly restored to room temperature and then 1.0 mL of a blood sample was added to the reagent. The tube was gently inverted and mixed five times, and 20 μL of 0.2 mol/L calcium chloride was added to the preheated reaction cup of the TEG instrument. Subsequently, 340 μL of the sample was drawn and mixed with kaolin in the reaction cup. The cup slot was moved up and the tests started.

### 2.3 Statistical analysis

Statistical descriptions of the routine coagulation function indicators (i.e., the PLT count and TEG parameters) in patients with benign and malignant breast diseases were determined. The data were normalized during the clustering analysis. After excluding data related to a number of complex cases of non-tumor diseases, the dataset (*n* = 120) is presented in **Supplementary Table S1** following a preliminary normalization treatment.

SPSS 25.0 statistical software was used to conduct general descriptive statistics on various indicators in the benign breast and malignant disease groups. A *t*-test was used to examine differences in the coagulation-related indices between the breast cancer group and the benign breast disease group, and the test values were statistically significant (*p* < 0.05). Correlation analysis was also performed on various indicators, such as the TEG, PLT count, and coagulation function in patients with breast diseases using Pearson’s correlation coefficient. The ROC curve was used to analyze the predictive value of TEG, routine PLT test, and coagulation function for the secondary hemorrhagic disease of patients with breast cancer.

## 3 Results

### 3.1 Descriptive statistics

The descriptive statistics of the routine coagulation function indicators, PLT count, and the TEG parameters in patients with benign (B) and malignant (M) breast diseases, are shown in **Table 2**. The timeline of the TEG curve from left to right can be divided into three stages: 1) from the activation of the coagulation factor to fibrin formation; 2) fibrin formation, PLT aggregation, and blood clot formation; and 3) fibrinolysis. Among the TEG parameters, the mean values of reaction time (*R*) and coagulation time (*K*) in the malignant breast disease group were lower than those in the benign breast disease group. These indicate the patients’ hypercoagulability or hyperfibrinolysis with consideration of the coagulation factor function (*R*), FIB function [*K* and the coagulation angle (*α*)], and PLT function (maximum amplitude, MA) in the whole blood. Of these parameters, *R* has indicative effects in the clinical evaluation of blood coagulation function, the guidance of component transfusion, and the prediction of thrombus/bleeding and medication risks.

**Table 2:** Results of the routine coagulation function indicators, platelet count, and thromboelastogram (TEG) indices and variations.

Item	Tumor	Normal	Mean	SD	Min.	Max.
*R* (min)	B	5–10	6.01	1.38	2.9	10.6
	M		5.80	1.57	3.2	10.5
*K* (min)	B	1–3	1.62	0.56	0.8	4.1
	M		1.51	0.48	0.9	2.9
*α* (deg)	B	53–72	67.05	6.10	41.4	78.4
	M		68.18	6.49	52.3	78.5
MA (mm)	B	50–70	61.78	9.00	−2.6	73.7
	M		64.74	4.47	56.1	75.5
CI (a.u.)	B	−3 to 3	0.50	1.93	−7.2	3.8
	M		0.95	2.02	−3.9	3.9
PT (s)	B	9–13	10.84	0.69	8.9	12.7
	M		11.05	0.79	9.7	13.0
aPTT (s)	B	23.3–32.5	26.17	2.32	21.4	35.6
	M		26.05	1.99	22.2	30.8
TT (s)	B	14–21	17.17	1.03	14.7	20.7
	M		17.23	0.96	14.6	19.1
FIB (g/L)	B	2–4	2.97	0.73	1.909	5.493
	M		3.50	1.10	2.044	7.590
D-D (mg/L)	B	<0.55	0.50	0.46	0.10	2.11
	M		1.56	4.75	0.10	26.70
PLT (×10^9^/L)	B	100–350	228.14	80.27	70	480
	M		236.36	65.95	116	401

R, reaction time; K, coagulation time; α, coagulation angle; MA, maximum amplitude; CI, coagulation index; PT, prothrombin time; aPTT, activated partial thromboplastin time; TT, thrombin time; FIB, fibrinogen; D-D, D-dimer; PLT, platelets; B, benign; M, malignant.

**Figure 2:**
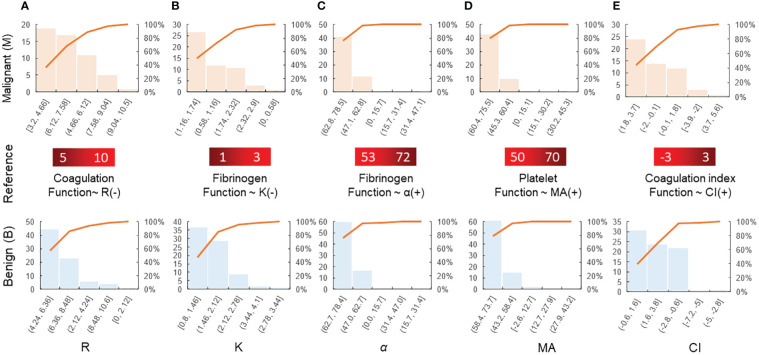
Histogram of various parameters derived from the thromboelastography maps of patients with benign/malignant breast tumors: **(A)** R, **(B)** K, **(C)** α, **(D)** MA, and **(E)** CI. The coagulation factor activity R (2–8min) reflects the function of clotting factors. Both K and α-angle reflect the function of fibrinogen. MA reflects the function of platelets. CI, coagulation composite index, is of great significance for the clinical prediction of thrombosis and bleeding. The blood sample is in a hypercoagulable state when CI>3, and the blood sample is in a hypocoagulable state when CI is less than −3.

The histograms for the group data distribution after processing for normalization and statistics are presented in **Figure 2**. There were significant differences in the statistical characteristics of the histograms among patients with benign and malignant breast tumors in some indices of the TEG atlas [e.g., *R*, *K*, and the coagulation index (CI)]. For patients with benign breast tumors, the *R* value had a higher frequency in the range 3.2–9.04, indicating that this value is not sensitive to the clinical reference of patients with benign breast tumors. On the other hand, for patients with malignant tumors, the *R* value was concentrated in the range 4.28–8.48, and the distribution density at both ends was low. Both malignant and benign breast tumors tend to obey normal distribution. Nevertheless, in comparison, the expected value of a malignant tumor was smaller (*µ* ≈ 5.8) and the mean variance was larger (*σ* ≈ 1.57). For patients with benign breast tumors, the *K* value was highly concentrated in the range 1.16–1.74, and its distribution on both sides was more uniform, tending to obey a normal distribution. The expected value *µ* was approximately 1.62, while the mean variance *σ* was approximately 0.56. For malignant breast tumors, the expected value *µ* was reduced to 1.51, while the mean variance *σ* was approximately 0.48.

**Figure 3:**
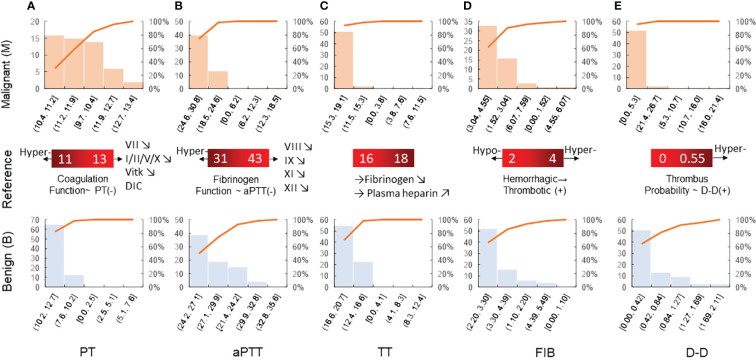
Histogram of blood routine coagulation indexes of patients with benign/malignant breast tumors: **(A)** PT, **(B)** aPTT, **(C)** TT, **(D)** FIB, and **(E)** D-D. Prothrombin time is the extrinsic coagulation pathway to reflect the extrinsic coagulation function. aPTT suggests the abnormal intrinsic coagulation factor and the screening test reflects the intrinsic coagulation pathway. Thrombin time is the required time for plasma coagulation after the standardized thrombin solution. Fibrinogen is the detection of plasma fibrinogen, and its increase is seen in thrombotic diseases and conversely in hemorrhagic diseases.

The mean values of the *α* angle, MA, and CI were higher in the malignant breast disease group. Of the coagulation function indicators, the mean values of aPTT and PLT in the malignant breast disease group were lower than those in the benign breast disease group. The histograms for the different variable distributions of patients with benign/malignant breast tumors are presented and compared in **Figure 3**. The mean values of PT, TT, FIB, and D-D were higher in the group of benign breast diseases. Comparison of the blood routine parameters revealed differences in the statistical characteristics of the histograms between benign and malignant breast tumors, particularly in PT and aPTT. The PT values of benign tumors were highly concentrated in the range 10.2–12.7, while those of malignant breast tumors were more widely distributed in the range 9.7–11.9. The expected PT value of malignant tumors is offset by 0.2 to the right, indicating an increased probability of anticoagulants in the blood. In addition, the expected value of FIB in the blood of patients with malignant breast tumors increased from 2.98 to 3.5. The FIB in the blood of patients with malignant tumors was significant, indicating an increased risk of cardiovascular disease.

### 3.2 Disparity level

Disparity level testing was conducted for the routine coagulation function indicators, PLT count, and the TEG parameters in patients with benign and malignant breast diseases. A *t*-test was used to examine differences in the routine coagulation function indicators, PLT count, and the TEG parameters in patients with benign and malignant breast diseases (**Table 3**).

**Table 3:** Differences in the routine coagulation function indicators.

Parameter	Homogeneity of variance		*t*-test	
	*F*	*p*	*t*	*p*
*R*	1.502	0.223	0.818	0.415
*K*	0.214	0.645	1.209	0.229
*α*	0.745	0.390	−1.015	0.312
MA	1.517	0.220	−2.206	0.029*
CI	0.671	0.414	−1.299	0.196
PT	3.097	0.081	−1.556	0.122
aPTT	0.245	0.622	0.291	0.771
TT	0.187	0.666	−0.332	0.740
FIB	2.134	0.146	−3.256	0.001*
D-D	10.348	0.002*	−1.608	0.114
PLT	1.058	0.306	−0.617	0.538

R, reaction time; K, coagulation time; α, coagulation angle; MA, maximum amplitude; CI, coagulation index; PT, prothrombin time; aPTT, activated partial thromboplastin time; TT, thrombin time; FIB, fibrinogen; D-D, D-dimer; PLT, platelets.

* read the statistical significance (*p*<0.05).

Differences in the routine coagulation function indicators, PLT count, and the TEG parameters were analyzed in patients with benign and malignant breast diseases. The statistical *t*-values and *p*-values of D-D were obtained without assuming equal variance conditions, while the *t*-values and *p*-values of the other indicators were obtained under assumption of equal variance conditions. Correlation analysis of the routine coagulation function indicators, PLT count, and TEG parameters was conducted in patients with breast diseases. The results of the homogeneity of variance showed that PT and D-D were statistically significant (*p* < 0.05). The results of normal fitting showed that the expected value of D-D in malignant breast tumors increased by 200%. D-D is generally prone to increase after thrombosis, which is an important molecular marker for its diagnosis.

**Figure 4:**
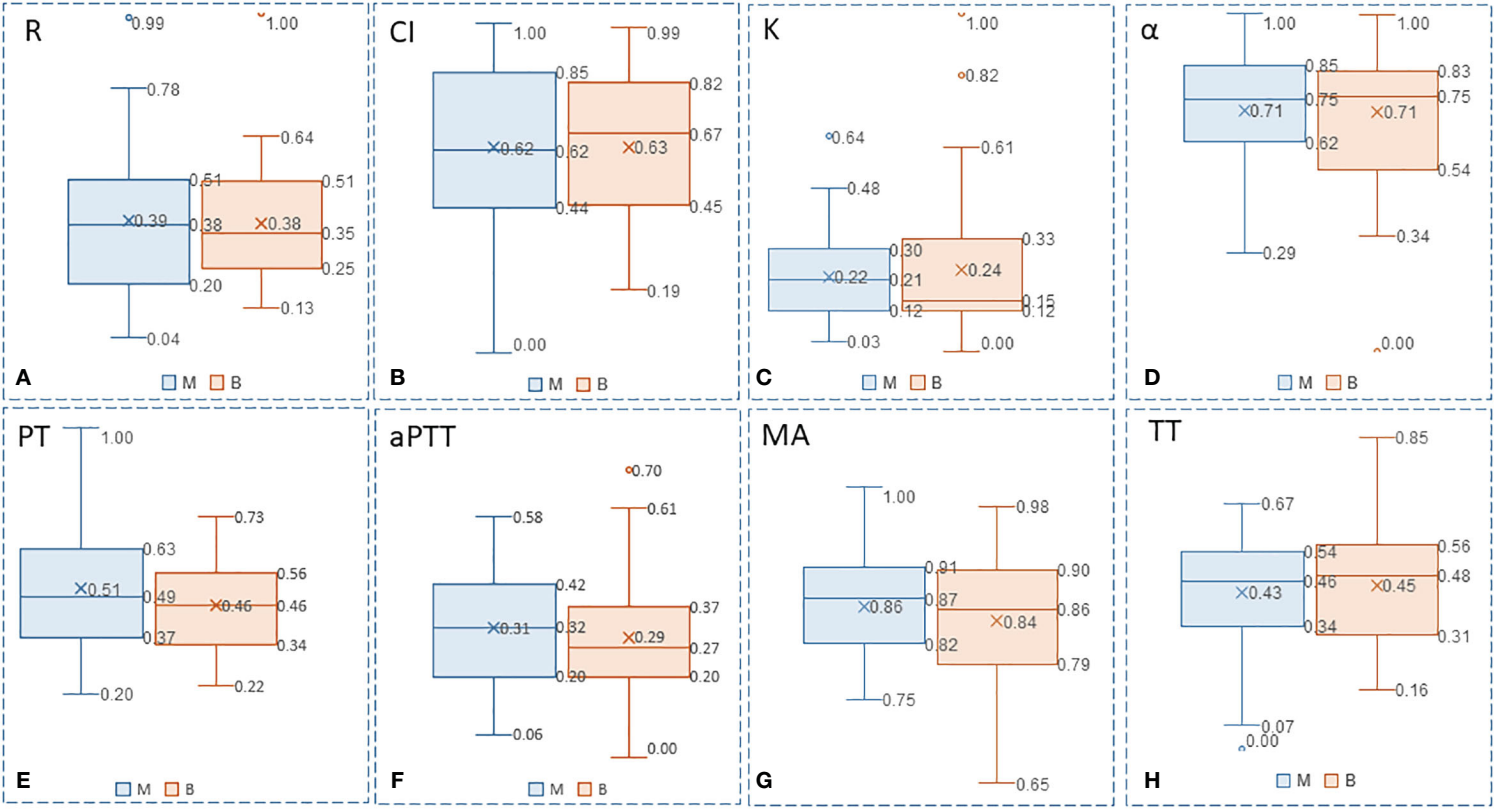
Box-whisker plots of the various blood transfusion indexes of patients with benign/malignant breast tumors: **(A)** R, **(B)** CI, **(C)** K, **(D)** α, **(E)** PT, **(F)** aPTT, **(G)** MA, and **(H)** TT. Five variables (Upper limit, RO 75% quantile, median, Rd 25% quantile, lower limit) were presented to construct box-whisker plots. The plots show the average level, degree of fluctuation, and outliers, and the width of the box reflects the degree of fluctuation of the data to some extent.

Increased values are common in deep venous thrombosis, pulmonary embolism, myocardial infarction, and hyperfibrinolysis secondary to DIC. The *t*-test for the MA value and the FIB index in the benign and malignant breast disease groups showed *p* < 0.05, with significant difference between the two groups. However, the *t*-test for the other indicators showed *p* > 0.05, and the differences were not statistically significant. Furthermore, the box–whisker plots of the various blood transfusion indices of patients with benign/malignant breast tumors were compared (**Figure 4**), which were in accordance with the results of the *t*-tests.

### 3.3 Correlation analysis

Correlation analysis of the given parameters is of clinical significance. In the determination of the TEG map parameters and the four coagulation tests, it was found that they are mostly prone to present variation trends in the blood coagulation state. Pearson’s correlation coefficient was used to analyze the correlation between TEG, routine PLT, and the coagulation function indicators in patients with breast diseases. The results of the correlation analysis of the medical function indicators and/or the parameters in patients with breast diseases are shown in **Table 4**.

**Table 4:** Correlation analysis of the routine coagulation data and TEG parameters.

		*R*	*K*	*α*	MA	CI
PT	*r*	−0.060	−0.016	0.016	0.082	0.077
	*p*	0.495	0.853	0.858	0.350	0.382
aPTT	*r*	0.208	0.250	−0.232	−0.116	−0.281
	*p*	0.017*	0.004**	0.008**	0.188	0.001**
TT	*r*	0.137	0.066	−0.056	−0.094	−0.115
	*p*	0.118	0.451	0.526	0.288	0.193
FIB	*r*	0.021	−0.080	−0.035	0.210	0.077
	*p*	0.809	0.365	0.689	0.016	0.386
D-D	*r*	0.210	0.266	−0.279	−0.085	−0.249
	*p*	0.016*	0.002**	0.001**	0.332	0.004**
PLT	*r*	−0.195	−0.341	0.320	0.279	0.336
	*p*	0.025*	0.000**	0.000**	0.001**	0.000**

R was positively correlated with aPTT and D-D, but was negatively correlated with PLT. K was positively correlated with aPTT and D-D, but was negatively correlated with PLT. The α angle was negatively correlated with aPTT and D-D, but was positively correlated with PLT. MA was positively correlated with FIB and PLT. CI was negatively correlated with aPTT, TT, and D-D, but was positively correlated with PLT.

R, reaction time; K, coagulation time; α, coagulation angle; MA, maximum amplitude; CI, coagulation index; PT, prothrombin time; aPTT, activated partial thromboplastin time; TT, thrombin time; FIB, fibrinogen; D-D, D-dimer; PLT, platelets.

*p < 0.05 (double tail); **p < 0.01 (single tail).

### 3.4 Sensitivity and specificity

The ROC curve or sensitivity curve is the core index of the performance evaluation of medical diagnostic tests and predictive models. Through analysis of the results of the disease group and the control group, the upper and lower limits, the group distance, and the cutoff points of the measured values were determined. A cumulative frequency distribution table was listed according to the selected group spacing interval. The true positive rate (TPR; sensitivity), the specificity, and the false-positive rate (FPR; 1 − specificity) of all cutoff points were calculated and plotted as the ROC curves. The abscissa and ordinate of the ROC curve were the non-specificity and sensitivity, respectively. The sensitivity and the specificity of the routine coagulation function indicators and the TEG parameters in detecting secondary bleeding disorders in patients with breast cancer were analyzed. The ROC curve was used to analyze the predictive value of TEG, routine PLT, and coagulation function indicators for secondary bleeding disorder in patients with breast cancer.

**Figure 5:**
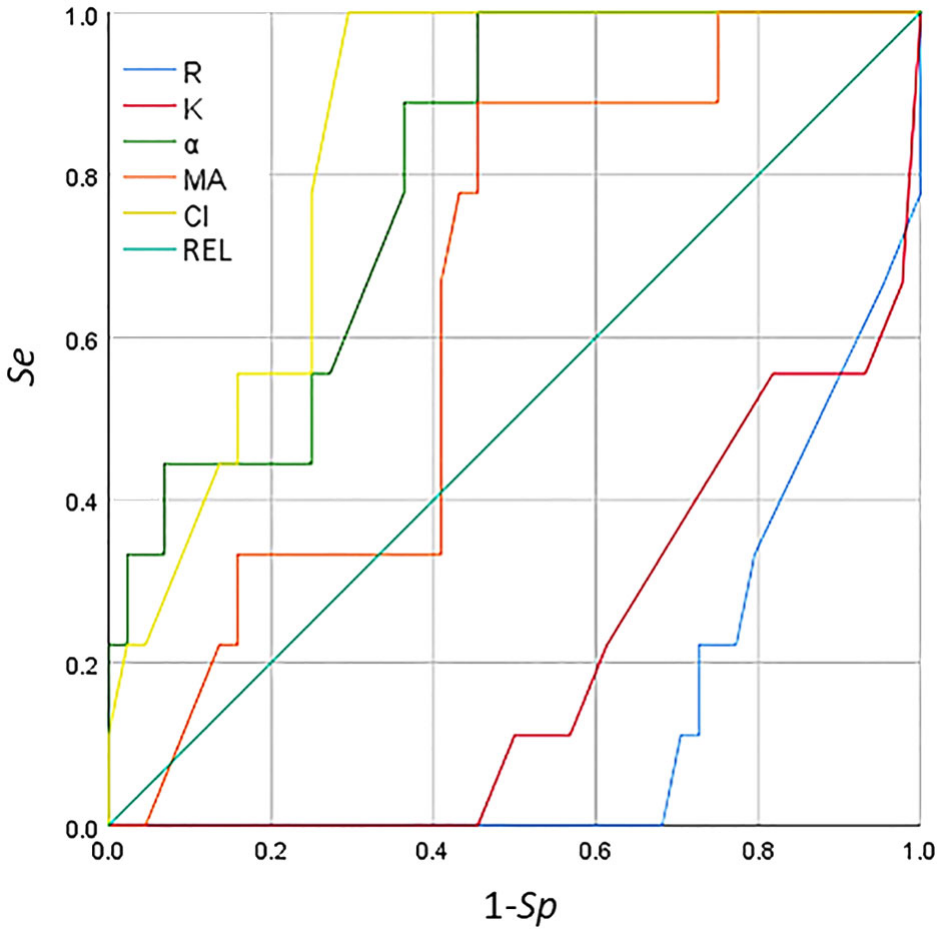
Receiver operating characteristic (ROC) curve analysis of the thromboelastogram (TEG) parameters for the detection of secondary bleeding disorders in patients with breast cancer.

The ROC curve can be used for threshold selection and model comparison; however, due to the limited number of samples, the ROC curves were not smooth but stepped. A change in the FPR (1 − Sp) and the TPR (Se) requires at least one sample to change. The area under the ROC curve (AUC) can be used for model comparison. Among the TEG parameters in the ROC curve (**Figure 5**), CI and the *α* angle showed significant predictive value for classification (AUC > 0.8) for detecting secondary bleeding disorders in patients with breast cancer. The diagnostic value of the other indices was fairly low (AUC < 0.7).

**Figure 6:**
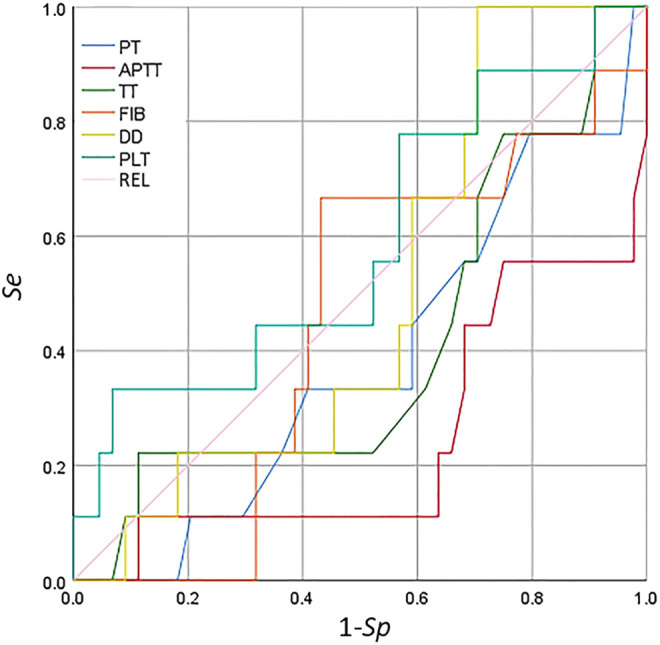
Receiver operating characteristic (ROC) curve analysis of the coagulation function indicators and platelet count for the detection of secondary bleeding disorders in patients with breast cancer.

The ROC curve of the coagulation function indicators and PLT is shown in **Figure 6**. The indices of aPTT, TT, PT, and DD were not in line with the actual scenario (AUC < 0.5), whereas PLT was not significant (AUC < 0.7) in detecting secondary bleeding disorders in patients with breast cancer. For the results of the coagulation function indicators and PLT, the correlation between each indicator and secondary bleeding disorder was not significant for the detection of bleeding disorders.

## 4 Discussion

### 4.1 Parameters in the thromboelastogram

Different from the conventional coagulation function index, a thromboelastogram can reflect the dynamic process of coagulation, including PLT aggregation (44), coagulation factor initiation (45), fibrin formation, and fibrinolysis (46). The clinical significance is not limited to thrombotic disease (47), thrombocytopenia, coagulation factor deficiency diseases, and hyperfibrinolytic disease. A series of parameters are derived and presented following TEG (48). The *R* value indicates the time required for the initiation of clotting and the formation of initial fibrin clots. In the TEG test, the intrinsic coagulation factors and anticoagulants affect the *R* value (49). With the continuous activation of the coagulation cascade reaction, the strength of blood clotting continuously increases. The *K* value refers to the time from the formation of the initial fibrin clot to a certain strength of blood clotting. This value is related to the PLT concentration and function, the extrinsic coagulation factor, thrombin, FIB, and hematocrit (46). An increase in *K* indicates that the function of FIB in the whole blood of the patient is insufficient, while a decrease in *K* indicates a hyperfunction of FIB. The *α* angle reflects the rate of clot formation, specifically the rate of fibrin formation and the cross-linking of fibrin and PLTs (50). The effect of the *α* angle is consistent with the reciprocal of *K* and is affected by the same factors. An increased *α* angle is related to an increased FIB function in the whole blood of patients, while a decreased *α* angle is related to insufficient FIB function (51). MA indicates the maximum clotting strength, while the PLT concentration function, the FIB concentration, and the PLT–FIB interaction all determine the clotting strength and the influence of PLTs (52). An increased MA indicates an increased PLT function in the whole blood of a patient, while a decreased MA indicates a decreased PLT function. The end of the clotting process is followed by the fibrinolysis process (53).

TEG tests can indicate various diseases related to perioperative bleeding of unknown origin, suspicion of hyperfibrinolysis, monitoring of FIB replacement, thrombocytopenia, and disorders of polymerization (54). The tests derive a series of parameters by combining relevant software processing in the TEG maps. The *R* value represents the time required for the initiation of coagulation and the formation of initial fibrin clots (55). The intrinsic coagulation factors and anticoagulants influence the *R* value, with an increased *R* indicating a low coagulation state, such as an increased coagulation factor activity and the use of anticoagulants (56). A reduced *R* indicates no fibrinolysis and that the blood is in a hypercoagulable state, such as a decrease in the coagulation factor activity for the PLT-rich plasma and in patients with chronic dissection (57). As the coagulation cascade reaction is continuously activated, the strength of blood clotting continues to increase (58). MA represents the maximum clotting strength reached by a blood clot. The PLT concentration and function, the FIB concentration, and the interaction of PLTs with FIB all determine the strength of blood clotting, with the influence of PLTs being major (59).

### 4.2 Blood coagulation function index

The coagulation indicators, including *R*, *K*, MA, and CI, are correlated with a diagnosis of thrombosis or embolism. In particular, decreased *R* and *K* values and increased *α* angle, MA, and CI indicate that the blood is prone to be in a hypercoagulable state. An increase in FIB and D-D is also closely related to a high coagulation state. Linkins and Lapner summarized the general characteristics of D-D detection, discussed the concept of increasing the threshold of D-D for the diagnosis of VTE according to prior probability and age, and provided a clinical point of view on the role of D-D detection in the diagnosis and prognosis of VTE (60). In the descriptive statistics of the routine coagulation function indicators, PLT count, and the TEG parameters in patients with benign and malignant breast diseases, the mean values of *R* and *K* in the malignant breast disease group were lower than those in the benign breast disease group, but the mean values of the *α* angle, MA, CI, FIB, and D-D were higher than those in the benign breast disease group. The results of the test of differences in the routine coagulation function indicators, PLT count, and the TEG parameters in patients with benign and malignant breast diseases showed that the *t*-test for MA and FIB in both benign and malignant breast disease groups was *p* < 0.05, indicating significant difference between the two groups. The indicators in patients with malignant breast diseases pointed to hypercoagulability, which differed somewhat from those in patients with benign breast diseases (61). Moreover, at 90% confidence interval, the FIB test had a *p* < 0.1, while the *p*-values of CI, D-D, PLT, and the other indicators tested for *p* > 0.1 were very close to 0.1. Correlation analysis of the routine coagulation function indicators, PLT count, and the TEG parameters in patients with breast diseases showed that the *R* value was positively correlated with aPTT and D-D, but was negatively correlated with PLT. The *K* value was positively correlated with aPTT and D-D, but was negatively correlated with PLT. The *α* angle was negatively correlated with aPTT and D-D, but was positively correlated with PLT. The MA was positively correlated with FIB and PLT. The CI was negatively correlated with aPTT, TT, and D-D, but was positively correlated with PLT. The correlation analysis between *R* and APTT (or PT) showed a higher response degree of kaolin-activated coagulation for intrinsic coagulation pathways than that of extrinsic coagulation pathways. PLT was negatively correlated with *R* and *K*, as well as the *α* angle. There was a positive correlation between the *α* angle, MA, and CI, indicating consistency between the TEG examination and the PLT count. The PLT concentration and function, the FIB concentration, and the interaction between PLTs and FIB all determine the strength of blood clotting. MA was positively correlated with FIB and PLT, which is consistent with this conclusion. ROC curve analysis of the predictive value of TEG, routine PLT, and the coagulation function indicators for secondary bleeding disorders in patients with breast cancer showed that CI and the *α* angle had predictive value. The CI and the *α* angle were obtained from the TEG testing parameters, indicating that TEG testing has significant predictive value for secondary bleeding disorders in patients with breast cancer. TEG detection for breast cancer patients is on the way, accompanied by routine coagulation assessment to prevent bleeding disease.

### 4.3 Blood hypercoagulability

The formation of blood hypercoagulability in patients with breast cancer increases the risk of thrombosis and hemorrhagic disease and promotes the occurrence and development of tumors. In addition, this hypercoagulable state supports tumor immune escape and interferes with immunotherapy. Blood hypercoagulability can be measured using various indices (e.g., CI, *R*, *K*, MA, PT, TT, aPTT, FIB, and D-D), despite these indices having clear differences among them when referring to normal ranges. Thromboelastographic detection should be carried out at the same time as routine coagulation function assessment in patients with breast cancer, which provides an important basis for rational clinical treatment. There is increasing research on the correlation between routine coagulation function indicator assessment and thromboelastographic detection in tumor diseases. More and more attention will be paid to thromboelastographic detection, and it will also continue to improve and develop. Combined with the assessment of routine coagulation function indicators, enough theoretical references have become more available to provide the basis for the auxiliary diagnosis and prevention of malignant tumors and their secondary diseases.

Despite the reduced *R* and K values in the malignant breast disease group, there was a significant difference in the MA and FIB between the benign and malignant breast disease groups in the current study. This suggests that PLT function and FIB are sensitive to the occurrence and development of breast tumors. There was no significant difference in the other indices (*p* > 0.05). In comparison, the mean values of *α*, MA, CI, FIB, and D-D in the malignant breast disease group were higher than those in the benign breast disease group. Despite the D-D levels increasing in almost all cases of acute thromboembolism, D-D can be detected at low levels in healthy individuals due to the conversion of small amounts of FIB into fibrin physiologically, which also increases with age (60). LY30 and LY60 represent the strength of blood clotting at 30 and 60 min after the beginning of fibrinolysis, respectively, reflecting the speed of clot dissolution (62), despite the no clear variation between LY30 and LY60 in our study. An increase in these values indicates hyperfibrinolysis, while a decrease indicates a weakened fibrinolysis (63). As discussed above, the controlling and/or regulatory factors of blood hypercoagulability are complex, and our current research found statistical correlations between them. In this study, the mean value of CI was within the normal range, despite a variation between 0.5 and 0.95 for the patients with benign and malignant breast tumors.

## 5 Conclusion

Female patients with breast cancer are more prone to complications with blood hypercoagulable state than those with benign breast diseases. The combination of thromboelastography tracing and routine blood coagulation indices might have a more guiding role in the treatment and prediction of secondary bleeding disorders in patients with breast cancer. The *R* and *K* values in the malignant breast disease group were lower than those in the benign breast disease group; however, the *α* angle, MA, CI, FIB, and D-D in the malignant breast disease group were higher than those in the benign breast disease group. The CI, *α* angle, and MA of TEG might predict secondary bleeding disorders in patients with breast cancer.

## Data availability statement

The original contributions presented in the study are included in the article/**Supplementary Material**. Further inquiries can be directed to the corresponding authors.

## Ethics statement

The studies involving humans were approved by Ethics Committee of Drug Clinical Trials in Affiliated Hospital of Jiangsu University. The studies were conducted in accordance with the local legislation and institutional requirements. The participants provided their written informed consent to participate in this study.

## Author contributions

QP: Conceptualization, Funding acquisition, Investigation, Methodology, Project administration, Supervision, Writing – original draft, Writing – review & editing. JZ: Formal analysis, Software, Visualization, Writing – original draft. XR: Resources, Validation, Writing – review & editing.
