# Supplementary material for: Thromboelastogram and coagulation function index: relevance for female breast cancer
Source: Front Oncol. 2024 Jul 17;14:1342439. doi: 10.3389/fonc.2024.1342439 (PMC11288955; doi:10.3389/fonc.2024.1342439)

**Title**: Thromboelastogram and coagulation function index: clinical relevance for female breast cancer

**Authors**: PENG Qiongle*, ZHU Jinmei, REN Xiaoling*

Table S1 Dimensionless values of current blood indexes given in this study


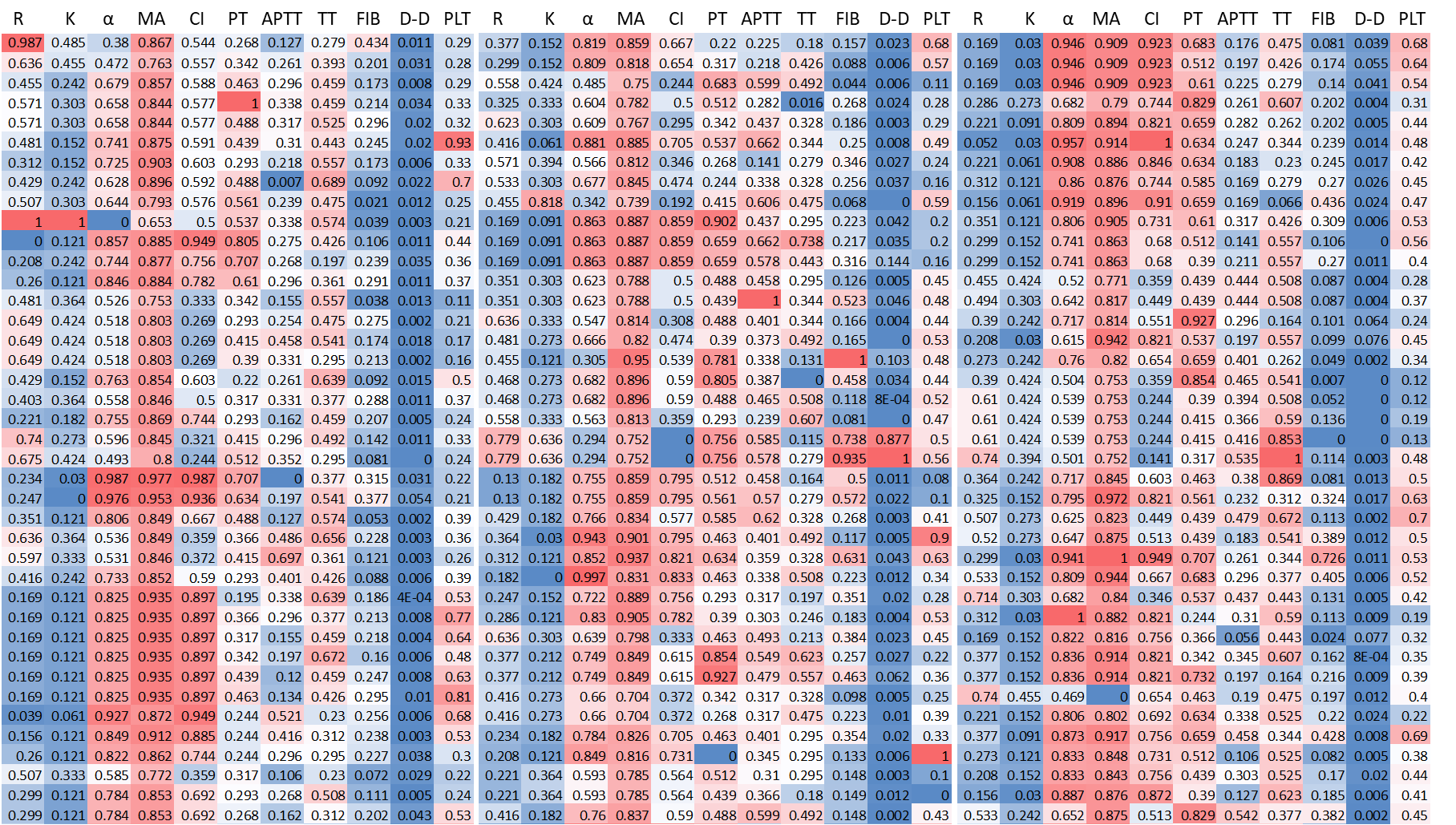

Supplement: Supplementary file 1 [file Table_1.docx]
